# Impact of obesity on survival in COVID-19 ARDS patients receiving ECMO: results from an ambispective observational cohort

**DOI:** 10.1186/s13613-021-00943-0

**Published:** 2021-11-15

**Authors:** Florence Daviet, Philippe Guilloux, Sami Hraiech, David Tonon, Lionel Velly, Jeremy Bourenne, Alizée Porto, Inès Gragueb-Chatti, Mickael Bobot, Karine Baumstarck, Laurent Papazian, Frédéric Collart, Jean-Marie Forel, Christophe Guervilly

**Affiliations:** 1 Medecine Intensive Réanimation, Centre hospitalier Universitaire L’Hôpital Nord, Assistance Publique hôpitaux de Marseille, chemin des Bourrely, 13015 Marseille, France; 2 Faculté de Médecine Centre d’Études et de Recherches sur les Services de Santé et qualité de vie EA 3279, Aix-Marseille Université, 13005 Marseille, France; 3 Département d’Anesthésie-réanimation, Centre hospitalier Universitaire La Timone, Assistance Publique Hôpitaux de Marseille, 13005 Marseille, France; 4 Médecine intensive Réanimation, Réanimation des Urgences, Centre hospitalier Universitaire La Timone 2, Assistance Publique Hôpitaux de Marseille, Marseille, France; 5 Département de chirurgie cardiaque, Centre Hospitalier Universitaire La Timone, Assistance Publique Hôpitaux de Marseille, Marseille, France; 6 Faculté de Médecine, Centre d’Etudes et de Recherches sur les Services de Santé et Qualité de Vie EA 3279, Aix-Marseille Université, Marseille, France

**Keywords:** COVID-19, ECMO, ARDS, Obesity, Prognosis

## Abstract

**Background:**

Since March 2020, health care systems were importantly affected by severe acute respiratory syndrome coronavirus 2 (SARS-CoV-2) outbreak, with some patients presenting severe acute respiratory distress syndrome (ARDS), requiring extra-corporeal membrane oxygenation (ECMO). We designed an ambispective observational cohort study including all consecutive adult patients admitted to 5 different ICUs from a university hospital. The main objective was to identify the risk factors of severe COVID-19 ARDS patients supported by ECMO associated with 90-day survival.

**Results:**

Between March 1st and November 30th 2020, 76 patients with severe COVID-19 ARDS were supported by ECMO. Median (interquartile range IQR) duration of mechanical ventilation (MV) prior to ECMO was of 6 (3–10) days. At ECMO initiation, patients had a median PaO_2_:FiO_2_ of 71 mmHg (IQR 62–81), median PaCO_2_ of 58 mmHg (IQR 51–66) and a median arterial pH of 7.33 (IQR 7.25–7.38). Forty-five patients (59%) were weaned from ECMO. Twenty-eight day, 60-day and 90-day survival rates were, respectively, 92, 62 and 51%. In multivariate logistic regression analysis, with 2 models, one with the RESP score and one with the PRESERVE score, we found that higher BMI was associated with higher 90-day survival [odds ratio (OR): 0.775 (0.644–0.934), *p* = 0.007) and 0.631 (0.462–0.862), respectively]. Younger age was also associated with 90-day survival in both models [OR: 1.1354 (1.004–1.285), *p* = 0.044 and 1.187 (1.035–1.362), *p* = 0.014 respectively]. Obese patients were ventilated with higher PEEP than non-obese patients and presented slightly higher respiratory system compliance.

**Conclusion:**

In this ambispective observational cohort of COVID-19 severe ARDS supported by ECMO, obesity was an independent factor associated with improved survival at 90-day.

**Supplementary Information:**

The online version contains supplementary material available at 10.1186/s13613-021-00943-0.

## Background

Since March 2020, Europe and France health care systems were importantly affected by severe acute respiratory syndrome coronavirus 2 (SARS-CoV-2) outbreak. Although most patients infected by coronavirus disease 2019 (COVID-19) present mild or moderate symptoms, about 10% will need hospitalization and 1.5% will require intensive care unit (ICU) hospitalization. Among them, around 70% need respiratory support by mechanical ventilation (MV) [1–4], and present with moderate to severe acute respiratory distress syndrome (ARDS).

In non-COVID-19 patients presenting with severe ARDS, extra-corporeal membrane oxygenation (ECMO) is a potentially life-saving strategy [5–8], when refractory hypoxemia or injurious mechanical ventilation parameters persists, despite low tidal volume ventilation associated with continuous neuromuscular blockers infusion and prolonged and repeated prone positioning. The initial reports of ECMO use in COVID-19 patients showed high mortality rates and raised concerns regarding the indication of the technique in this disease [9]. However, international organizations (ELSO, ECMOnet) recommended and proposed ECMO guidelines for COVID-19 ARDS patients, with similar indications as for other ARDS etiologies based on EOLIA criteria [10–12]. Of note, ECMO support may be no more indicated in case of overwhelmed health care system. Rapidly, numerous cohorts with high number of patients and similar outcomes as non-COVID-19 ARDS supported by veno-venous ECMO (vvECMO) were published [13–16]. These results are important as the worldwide pandemic justifies optimal utilization of scarce resources, and the use of treatment or techniques that would secondarily appear futile should be avoided.

To better identify the population of patients that could benefit from the technique, we planned to identify the prognostic factors associated with survival in severe COVID-19 ARDS patients supported by ECMO.

## Materials and methods

### Study design and setting

We conducted a single-center ambispective observational cohort study including all consecutive adult patients admitted to the tertiary University Hospital of Assistance Publique-Hôpitaux de Marseille France, constituted of 5 different ICU units, with a diagnosis of confirmed COVID-19 associated pneumonia, documented by at least one real-time RT PCR test in nasopharyngeal swabs, and supported by ECMO for severe ARDS.

Among the 5 ICUs of our institute, one is the referral ECMO center of an area of 5 million inhabitants, has an ECMO mobile team available 24 h per day to cannulate and bring back to our hospital all patients from the referring hospitals. We used previously published criteria to indicate ECMO support [6].

The inclusion period covered the two first waves of the outbreak: from March 1st 2020 to May 31th 2020 for the first wave, and from September 1st 2020 to November 30th 2020 for the second wave.

This study was approved by the local Ethics Commission (2020-53) and the French Society of Anesthesia and Critical Care (00010254-2020-262). According to French law, the informed consent was not required due to the design of the study.

### Data collection and outcomes

For the retrospective part of the study (first wave) data were collected through patients’ medical files, during the second wave the data were collected prospectively. We collected demographic characteristics including body mass index (BMI), comorbidities evaluated through Charlson score calculation, clinical presentation at ICU admission and severity scores [Simplified Acute Physiology score (SAPS II), Sequential Organ Function (SOFA)]. Specific treatments for COVID-19 and ICU therapies before ECMO support were also reported. When patients were transferred from referring hospitals by the ECMO mobile team, these data were retrieved from the initial ICU. Obesity was defined as a BMI superior or equal to 30 kg/m^2^. Ventilatory parameters, last blood-gas values before ECMO support were also collected, and we calculated for each patient different validated scores to predict mortality in ECMO patients: the RESP score [17] (Respiratory Extracorporeal Membrane Oxygenation Survival Prediction Score), the ECMO Net score [18], the PRESERVE score [19] (PRedictiong dEath for SEvere ARDS on veno-venous ECMO), the SOFA (Sequential Organ Function) score. Patients were then followed for 90 days since the first day of ICU admission and the following outcomes were collected: survival at day 28, day 60 and day 90, as well as date of ECMO weaning, MV duration, length of stay in ICU and in hospital. The occurrence of severe bleeding (defined by need of ≥ 2 red packed cells over 24 h), severe hemolysis (requiring ECMO circuit change), complications during the ECMO support (ventilatory-acquired pneumonia, bacteremia and septic shock, heparin-induced thrombocytopenia, arterial or venous thrombosis) and during the ICU stay [need for renal replacement therapy, reactive hemophagocytic syndrome defined according to the Saint-Antoine’s score [20], viral reactivations defined by positive viral load detected by PCR of herpes simplex virus (HSV) and cytomegalovirus (CMV), and invasive aspergillosis] were recorded.

### Statistical analysis

Data are expressed as median and interquartile range (IQR) or numbers and percentage (%). We compared demographics data and pre-ECMO characteristics according to the 90-day post-ICU admission survival. Continuous variables were compared using the Student’s *T*-test. Categorical variables were compared using the Chi-square or Fisher’s exact tests. After testing collinearity, variables with *p* values < 0.1 in univariate analysis were entered in the multivariate logistic regression model. Results are expressed as odds ratios (OR) with 95% confidence intervals, and *p* values. The overall fit of the model was evaluated by Hosmer–Lemeshow test. We used two multivariate models, one for each predictive survival score under ECMO, to avoid collinearity as some variables are common in both scores. Independent prognostic factors of 90-day survival identified by multivariate logistic regression were used to dichotomize population and to construct Kaplan–Meier cumulative survival curves. Then, curves were compared with the log-rank test. We also split our cohort in two groups, obese and non-obese patients, and compared patients’ characteristics, severity and ECMO prognosis scores, as well as treatments received, delay from ICU admission to intubation and to ECMO therapy, respiratory mechanics and blood-gas values before ECMO. 73 patients were included in this analysis, 3 patients were excluded because of missing data for BMI. All statistical analyses were done using SPSS (IBM SPSS Statistics 20).

## Results

Between March 1st and November 30th 2020, 76 patients with severe COVID-19 ARDS were supported by ECMO, 77 (99%) by vvECMO and 1 patient by veno-arterial ECMO. Twenty-three patients (30%) were admitted during the first wave and 53 (70%) during the second wave. Patients’ main characteristics are presented in Tables 1 and 2. Concerning specific treatments, all patients of the second wave received dexamethasone at the dosage of 6 mg per day or equivalent doses of corticosteroids, but only one patient of the first wave, following the national management recommendations for SARS CoV-2 pneumonia [21]: overall, 67% of the cohort received dexamethasone or equivalent.

**Table 1:** Characteristics of the cohort and outcomes

	76 patients
**Patient ‘s characteristics**	
Comorbidities, *n* (%)	
Obesity^a^	29 (38%)
Hypertension	32 (42.1%)
Diabetes	28 (36.8%)
Coronary artery disease	9 (11.8%)
Dyslipidemia	16 (21.1%)
Smoking	15 (19.7%)
Immunosuppression	5 (6.6%)
Chronic lung disease	9 (11.8%)
Chronic kidney disease	4 (5.3%)
Number of comorbidities	0 (0–1)
Type of ECMO support, *n* (%)	
Veno-venous	75 (98.7%)
Arteriovenous	1 (1.3%)
Cardiac arrest before ECMO, *n* (%)	4 (5.3%)
** Outcome**	
ECMO weaning, *n* (%)	45 (59.2%)
ECMO second run, *n* (%)	5 (6.6%)
ECMO-free days at D60	14 (0–36.5)
MV duration	41 (27.5–54.5)
Mortality, *n* (%)	
Day 28	6 (7.9%)
Day 60	29 (38.2%)
Day 90	37 (48.7%)
Length of stay in ICU, (days)	47.5 (33–65)
Length of stay in hospital, (days)	50 (38–80)
**Complications** **during** **ICU** **stay,** ***n*** **(%)**	
Invasive aspergillosis	4 (5.3%)
Viral reactivation	
CMV	32 (43.2%)
Antiviral treatment	30 (94%)
HSV	21 (28.4%)
Antiviral treatment	15 (71%)
**Cause of death,** ***n*** **(%)**	
Septic shock	13 (17.1%)
Hemorrhagic shock	12 (15.8%)
Cardiogenic shock	1 (1.3%)
Stroke	2 (2.6%)
Multi-organ failure	3 (3.9%)
Other	6 (7.9%)

Data are expressed as median (interquartiles) or number (percentage, %)

Obesity was defined by a BMI superior or equal to 30 g/m^2^

*BMI* body mass index, *NSAID* non-steroidal anti-inflammatory drug, *ACE* angiotensin converting enzyme, *ARB* angiotensin II receptor blockers, *SAPS II* Simplified Acute Physiology score, *SOFA* Sequential Organ Function, *ICU* intensive care unit, *ECMO* extra-corporeal membrane oxygenation, *MV* mechanical ventilation, *CMV* cytomegalovirus, *HSV* herpes simplex virus

^a^3 patients had missing data for BMI

**Table 2:** Comparison of patient’s characteristics according to their survival at day 90 in univariate analysis

	All (*n* = 76)	Survivors at D90 (*n* = 39)	Non-survivors at D90 (*n* = 37)	*p* value
**Demographic characteristics**				
Age (years)	61 (54–64.5)	58 (49–62)	63 (59–66)	< 0.001
Male sex, *n* (%)	59 (77.6)	30 (77)	29 (78)	0.88
BMI (kg/m^2^)	28.1 (26.1–31.8)	30.5 (27.2–35)	27.2 (25.6–29.9)	0.002
Obesity^a^	29 (39.7%)	21 (53.8%)	8 (23.5%)	0.008
ECMO Referral Center, *n* (%)	42 (55)	23 (59)	19 (51)	0.50
Transfer from referring hospital by ECMO mobile team	33 (43)	19 (49)	14 (38)	0.34
**Characteristics of Sars-CoV 2 pneumonia and treatments**				
First wave, *n* (%)	23 (30)	18 (46)	5 (13.5)	0.002
Time from first symptoms to ICU admission, (days)	7 (5.5–10)	5 (4–7)	6 (4–7)	0.43
Time from ICU admission to intubation, (days)	3 (0–6)	2 (0–5)	4 (1–7)	0.02
Sars-CoV 2 treatment				
Dexamethasone^b^ *n* (%)	54 (67)	21 (54)	33 (92)	< 0.001
Hydroxychloroquine, *n* (%)	24 (32)	17 (44)	7 (19)	0.02
Antiviral treatment, *n* (%)				
Lopinavir/ritonavir	3 (4)	3 (8)	0 (0)	0.08
Immunomodulatory treatment, *n* (%)				
Anti-IL 6 treatment	6 (8)	4 (10)	2 (5)	0.43
Anti- IL 1 treatment	12 (16)	8 (20.5)	4 (11)	0.25
Janus kinase 1/2 inhibitor	14 (18)	8 (20.5)	6 (16)	0.63
High-dose corticosteroid	47 (62)	22 (56)	25 (68)	0.32
**ICU admission scores**				
Charlson score	2 (1.5–3)	2 (1–3)	3 (2–3)	0.003
SAPS II	30 (27–36)	29 (23–36)	31 (27–38.5)	0.42
SOFA	3 (2–4)	3 (2–4)	3 (2–5)	0.76
**ICU therapy before ECMO**				
High-flow nasal O_2_, *n* (%)	67 (88)	32 (82)	35 (95)	0.09
Duration (days)	3 (1–6)	2.5 (1–6)	4 (1–7)	0.14
Non-invasive ventilation, *n* (%)	37 (49)	17 (44)	20 (54)	0.36
Duration (days)	1 (0–6)	1 (0–6)	1 (0–6)	0.70
Almitrine, *n* (%)	12 (16)	6 (16)	6 (17)	0.92
Inhaled nitric oxide, *n* (%)	53 (70)	28 (72)	25 (68)	0.69
Prone positioning, *n* (%)	75 (99)	38 (97)	37 (100)	0.33
Number of sessions	2 (1–4)	2 (1–4)	2 (1–4)	0.85
MV duration before ECMO	6 (3–10)	5 (3–9)	7 (3–11)	0.43
**Ventilation parameters before ECMO**				
FiO_2_	100 (100–100)	100	100	0.58
Plateau pressure, (cmH_2_O)	28 (25–30)	28 (24–31)	28 (26–30)	0.71
Positive end-expiratory pressure, (cmH_2_O)	12 (9.5–14)	12 (10–14)	12 (9–14)	0.38
Tidal volume, (mL/kg predicted bodyweight)	5.8 (5.1–6.2)	5.7 (5–6.1)	6.04 (5.3–6.3)	0.15
Respiratory rate, (breath per minute)	27 (23–30)	26 (23–30)	28 (21–30)	0.92
Mechanical power, (L/min)	19.7 (15.7–24)	19.4 (15.8–22.4)	19.99 (14.07–24.34)	0.74
Ventilatory ratio	2.42 (1.71–2.84)	2.32 (1.79–2.84)	2.42 (1.55–2.88)	0.90
Static compliance, (mL/cmH_2_O)	23 (16.7–28.6)	23.3 (15.2–30.8)	22.7 (20–27.3)	0.63
**Last blood-gas values before ECMO**				
PaO_2_/FiO_2_ ratio	71.5 (62–81)	72.5 (60–83)	71.5 (65.5–80)	0.97
pH	7.33 (7.25–7.38)	7.31 (7.25–7.39)	7.35 (7.25–7.37)	0.73
PaCO_2_, (mmHg)	58 (51–66)	58 (52–66)	58 (49–66)	0.85
**Predictive survival scores under ECMO**				
RESP score	1 (0–2)	1 (1–4)	1 (0–2)	0.01
ECMO net score	5 (4–6)	4.5 (4–6)	5 (4–6.25)	0.79
PRESERVE score	3 (2–4)	2 (1–4)	3 (3–5)	< 0.001
Last SOFA before ECMO	7 (4–9)	7 (4–10)	7 (4.5–9)	0.94
Respiratory component of SOFA	4 (4–4)	4 (4–4)	4 (4–4)	0.33
Cardiovascular component of SOFA	2 (0–4)	3 (0–4)	1 (0–4)	0.62
Hematological component of SOFA	0 (0–0	0 (0–0)	0 (0–0)	0.55
Renal component of SOFA	0 (0–1)	0 (0–0)	0 (0–0)	0.09
Time from ICU admission to ECMO, (days)	10.5 (7–13)	9 (4–11)	11 (8–14)	0.02
ECMO assistance duration, (days)	18 (11–31.5)	14 (9–23)	27 (15–39)	< 0.001
Prone positioning during ECMO, *n* (%)	61 (80)	33 (85)	28 (76)	0.33
Number of sessions	2 (1–4)	2 (1–4)	3 (0.5–5)	0.51
**Outcomes**				
ECMO weaning, *n* (%)	45 (59)	38 (97)	7 (19)	< 0.001
MV duration (days)	41 (27.5–54.5)	41 (29–57)	41 (25–53)	0.23
Complications occurred during the ECMO period, *n* (%)				
Intravascular hemolysis	34 (45)	14 (37)	20 (54)	0.13
Severe bleeding	42 (57)	11 (29)	31 (86)	< 0.001
Clogged circuit	11 (15)	6 (16)	5 (14)	0.82
Infection				
Ventilatory-acquired pneumonia	45 (61)	19 (50)	26 (72)	0.05
Bacteremia	33 (44)	16 (42)	17 (46)	0.74
Septic shock	45 (61)	16 (36)	29 (64)	0.001
CMV reactivation	32 (43)	13 (34)	19 (53)	0.16
CMV pneumonia	22 (29)	9 (23)	13 (35)	0.25
HSV reactivation	21 (28)	9 (24)	12 (33)	0.44
HSV pneumonia	14 (18)	6 (15)	8(22)	0.56
Arterial or venous thrombosis	36 (47)	27 (69)	9 (24)	< 0.001
Deep vein thrombosis or pulmonary thrombosis	34 (45)	26 (67)	8 (22)	< 0.001
Heparin-induced thrombocytopenia	3 (4)	3 (8)	0	0.08
Circuit change	30 (39)	13 (33)	17 (46)	0.26
Number of circuit change	0 (0–1)	0 (0–1)	0 (0–1)	0.02
Renal replacement therapy	25 (33)	8 (20)	17 (68)	0.02

Data are expressed as median (interquartiles) or number (percentage, %). Obesity was defined by a BMI superior or equal to 30 g/m^2^

Mechanical power (MP) was calculated as follows: MP = 0.098 × tidal volume × respiratory rate × (peak pressure − ½ × driving pressure). Driving pressure was defined as plateau pressure minus positive end-expiratory pressure. Static compliance was defined as tidal volume divided by driving pressure

BMI: body mass index; NSAID: non-steroidal anti-inflammatory drug; ACE: angiotensin converting enzyme; ARB: angiotensin II receptor blockers; SAPS II: Simplified Acute Physiology score; SOFA: Sequential Organ Function; ICU: intensive care unit; ECMO: extra-corporeal membrane oxygenation; MV: mechanical ventilation; O_2_: oxygen; PaO_2_: partial pressure of arterial oxygen; FiO_2_: fraction of Inspired oxygen; PaCO_2_: partial pressure of arterial carbon dioxide; PaO_2_/FiO_2_: ratio of the partial pressure of arterial oxygen to the fraction of inspired oxygen; RESP score [17]: Respiratory Extracorporeal Membrane Oxygenation Survival Prediction Score; ECMO net score [18]: score from the Italian ECMO network; PRESERVE score [19]: PRedicting dEath for SEvere ARDS on VV-ECMO score

^a^3 patients had missing data for BMI

^b^Patients receiving dexamethasone 6 mg per day or equivalent dose of corticosteroids

Median time from ICU admission to ECMO initiation was 10.5 days (7–13) and median duration of MV prior to ECMO of 6 days (3–10). Of note, 75 patients (98.7%) had at least one session of prone positioning before ECMO. At ECMO initiation, patients had a median PaO_2_:FiO_2_ of 71 (62–81) mmHg, PaCO_2_ of 58 mmHg (51–66) and arterial pH of 7.33 (7.25–7.38). Before ECMO, patients were ventilated in volume-controlled mode with a positive end-expiratory pressure (PEEP) of 12 (9.5–14) cmH_2_O, a plateau pressure (Pplat) of 28 (25–30) cmH_2_O for a tidal volume (TV) of 5.8 (5.1–6.2) mL/kg of predicted body weight. Respiratory system static compliance was 23 (16.7–28.6) mL/cmH_2_O.

Forty-five patients were weaned from ECMO. Among them, five patients (11%) required a second ECMO run. ECMO final weaning rate was 59%, 7 patients (15.5%) died after decannulation. ECMO duration was 18 (11–31.5) days. Twenty-eight day, 60-day and 90-day survival rates were, respectively, 92, 62 and 51%. Main causes of death were septic shock (35%), hemorrhagic shock (32%) and intractable respiratory failure (16%). Complications occurring during the ECMO support are described in Tables 1 and 2.

Univariate analysis regarding 90-day survival is provided in Table 2. Factors associated with 90-day survival were younger age, higher BMI and lower Charlson score. Of note, time from ICU admission to intubation [2 days (0–5) in survivors versus 4 days (1–7) in non-survivors, *p* = 0.02] and time to ECMO cannulation [9 days (4–11) in survivors versus 11 (8–14) in non-survivors, *p* = 0.02] were shorter in 90-day survivors.

Patients from the first wave had a better prognosis [78.2% survivors at day 90 among patients from the first wave versus 39.6% among patients from the second wave, *p* = 0.002]. We did not find any difference in ventilatory parameters before ECMO between the survivors and the non-survivors. Concerning the predictive survival scores, the RESP score and the PRESERVE score were significantly different between survivors and non-survivors at day 90.

To determine the independent factors predicting mortality at day 90, we used two models in multivariate logistic regression analysis: model A with the RESP score and model B with the PRESERVE score because of collinearity between those variables (*r* = − 0.598, *p* < 0.001) (Table 3). Only BMI and age in the two models were associated with 90-day survival.

**Table 3:** Multivariate analysis of predictors of 90-day mortality

	Odds ratio	95% CI	*p* value
**Model A**			
Age	1.135	1.004–1.285	0.044
BMI^a^	0.775	0.644–0.934	0.007
Charlson score	1.220	0.593–2.510	0.589
High-flow nasal oxygen before intubation	0.963	0.075–12.357	0.977
First wave	0.669	0.03–14.698	0.799
RESP score	1.117	0.673–1.853	0.669
Dexamethasone use^a^	5.211	0.375–72.412	0.219
Hydroxychloroquine use	0.673	0.108–4.186	0.671
Time from ICU admission to ECMO	1.108	0.951–1.291	0.187
**Model B**			
Age	1.187	1.035–1.362	0.014
BMI^a^	0.631	0.462–0.862	0.004
Charlson score	1.482	0.753–2.917	0.255
High-flow nasal oxygen before intubation	1.674	0.132–21.228	0.691
First wave	0.817	0.03–21.912	0.904
PRESERVE score	0.508	0.242–1.067	0.074
Dexamethasone use^b^	4.942	0.308–79.223	0.259
Hydroxychloroquine use	0.499	0.074–3.361	0.475
Time from ICU admission to ECMO	1.175	0.984–1.403	0.075

Model A: variables included: (a) quantitative: age, BMI, RESP score, Charlson score, time from ICU (intensive care unit) admission to ECMO; (b) qualitative: pandemic waves (first vs. second), dexamethasone use, hydroxychloroquine use, high-flow nasal O_2_ before intubation. Hosmer–Lemeshow test *p* = 0.558

Model B: variables included: (a) quantitative: age, BMI, PRESERVE score, Charlson score, time from ICU (intensive care unit) admission to ECMO; (b) qualitative: pandemic waves (first vs. second), dexamethasone use, hydroxychloroquine use, high-flow nasal O_2_ before intubation. Hosmer–Lemeshow test, *p* = 0.182

*BMI* body mass index (g/m^2^), *CI* confidence interval, *PRESERVE score* [19] PRedicting dEath for SEvere ARDS on VV-ECMO score, *RESP score* [17] Respiratory Extracorporeal Membrane Oxygenation Survival Prediction Score

^a^3 patients had missing data for BMI

^b^Patients receiving dexamethasone 6 mg per day or equivalent dose of corticosteroids

**Fig. 1:**
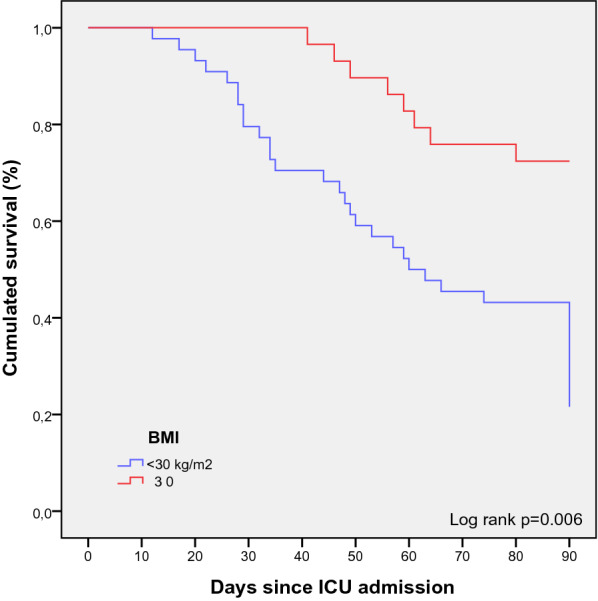
Kaplan–Meier cumulated survival curves at day 90 since ICU admission in obese (red curve) and non-obese patients (blue curve). Obesity was defined as a BMI ≥ 30 kg/m^2^

29 patients were obese in this cohort, with a higher proportion of obese patients in the survivors at day 90 [21 patients (53.8%) versus 8 patients (23.8%), *p* = 0.008]. These data are shown in Table 2. The median BMI of obese patients among the survivors at day 90 was 34.8 kg/m^2^ (31.1–46.9), which is higher from the median BMI of the whole survivors at day 90 which is 30.5 kg/m^2^ (24.2–35): there was 52% of patients with grade 1 obesity, 43% with grade 2 obesity and 5% with grade 3. Kaplan–Meier cumulated survival curves according to the presence of obesity were significantly different (Fig. 1, p = 0.006), the survival was also different when comparing the different obesity grades (Additional file 1: Fig. S1, *p* = 0.003).

Therefore, we compared obese and non-obese patients’ characteristics (Table 4). Obese patients were ventilated with higher PEEP than non-obese patients [14 (10–15) vs. 10 (8–12) cmH_2_O; *p* < 0.001] with comparable Pplat, 29 (25–31) vs. 28 (25–30) cmH_2_O, respectively (*p* = 0.923) and a trend to higher compliance of respiratory system in obese patients as compared with non-obese patients, respectively, 26.2 (21–39.1) and 23 (15.5–27.3) mL/cmH_2_O (*p* = 0.07). Obese patients had also a shorter delay from ICU admission to intubation (2 (0–4) days versus 4 (1–7) days in non-obese patients, *p* = 0.048) and to ECMO implantation [9 (4–11) days versus11 (8–14.5), *p* = 0.02]. Of note, obese patients were subjected before ECMO implantation, to a slightly higher mechanical power as compared with the non-obese patients, respectively, 20.3 (17.7–29.7) J/min and 17.7 (14.07–22.22) J/min (*p* = 0.06).

**Table 4:** Comparison of patient’s characteristics according to their obese status in univariate analysis

	Non-obese patients (*n* = 44)	Obese patients (*n* = 29)	*p* value
**Demographic characteristics**			
Age (years)	60.5 (56–64.5)	61 (54–64)	0.78
Sex (male)	35 (79.5%)	21 (72.4%)	0.48
BMI (kg/m^2^)	26.45 (25.35–27.8)	33.5 (31–35.8)	< 0.001
First wave, *n* (%)	16 (36)	7(24)	0.27
**Severity scores**			
SAPS II	30 (25.5–36)	29 (25.5–34.5)	0.60
SOFA	3 (2–4)	3 (2–4)	0.95
Charlson score	2 (2–3)	2 (1–3)	0.58
**ICU therapy before ECMO**			
High-flow nasal O_2_, *n* (%)	38 (86)	26 (90)	0.68
Duration (days)	5 (1–7)	2 (1–6)	0.08
Non-invasive ventilation, *n* (%)	18 (41)	17 (59)	0.14
Duration (days)	4 (0–6)	1 (0.5–3)	0.15
Prone positioning, *n* (%)	44 (100)	28 (97)	0.21
Number of sessions	2 (1–5)	2 (1.5–4)	0.88
Dexamethasone^a^, *n* (%)	28 (65)	19 (65.5)	0.97
Time from first symptoms to intubation, days	11 (7.5–15)	10 (7–13)	0.1
Time from hospitalization to intubation, days	5.5 (1–9)	4 (1–6)	0.06
Time from ICU admission to intubation, days	4 (1–7)	2 (0–4)	0.048
**Predictive survival scores under ECMO**			
RESP score	1 (0–2)	1 (1–4)	0.09
ECMO net score	5.5 (4–6)	4.5 (4–6)	0.73
PRESERVE score	4 (3–5)	2 (1–3)	< 0.001
SOFA before ECMO	8 (4–9)	5 (4–8)	0.58
MV duration before ECMO, days	7 (3–10.5)	5 (3–8)	0.35
Time from ICU admission to ECMO, days	11 (8–14.5)	9 (4–11)	0.02
**Ventilation parameters before ECMO**			
FiO_2_	100	100	0.58
Plateau pressure, cmH_2_O	28 (25–30)	29 (25–31)	0.92
Positive end-expiratory pressure, cmH_2_O	10 (8–12)	14 (10–15)	< 0.001
Driving pressure	17 (14–20)	14.5 (12–20)	0.1
Tidal volume, mL/kg predicted bodyweight	5.7 (5–6,21)	5.85 (5.6–6.2)	0.30
Respiratory rate, breath per minute	26 (23–30)	26.5 (20–30)	0.83
Mechanical power, J/min	17.7 (14.07–22.22)	20.3 (17.7–29.7)	0.06
Ventilatory ratio	2.42 (1.55–2.90)	2.34 (1.87–2.75)	0.81
Static compliance, mL/cmH_2_O	23 (15.5–27.33)	26.25 (21–39.09)	0.07
**Last blood-gas values before ECMO**			
PaO_2_/FiO_2_ ratio	71.5 (64–84)	75 (62–81)	0.93
pH	7.35 (7.26–7.38)	7.31 (7.26–7.39)	0.49
PaCO_2_, mmHg	57.5 (50.5–65.5)	60 (52–66)	0.99

73 patients were included in this analysis, 3 patients were excluded because of missing data for BMI

Data are expressed as median (interquartiles) or number (percentage, %)

Obesity was defined by a BMI superior or equal to 30 g/m^2^

Mechanical power (MP) was calculated as follows: MP = 0.098 × tidal volume × respiratory rate × (peak pressure − ½ × driving pressure). Driving pressure was defined as plateau pressure minus positive end-expiratory pressure. Static compliance was defined as tidal volume divided by driving pressure

BMI: body mass index; NSAID: non-steroidal anti-inflammatory drug; ACE: angiotensin converting enzyme; ARB: angiotensin II receptor blockers; SAPS II: Simplified Acute Physiology score; SOFA: Sequential Organ Function; ICU: intensive care unit; ECMO: extra-corporeal membrane oxygenation; MV: mechanical ventilation; O_2_: oxygen; PaO_2_: partial pressure of arterial oxygen; FiO_2_: fraction of Inspired oxygen; PaCO_2_: partial pressure of arterial carbon dioxide; PaO_2_/FiO_2_: ratio of the partial pressure of arterial oxygen to the fraction of inspired oxygen; RESP score [17]: Respiratory Extracorporeal Membrane Oxygenation Survival Prediction Score; ECMO net score [18]: score from the Italian ECMO network; PRESERVE score [19]: PRedicting dEath for SEvere ARDS on VV-ECMO score

^a^Patients receiving dexamethasone 6 mg per day or equivalent dose of corticosteroids

## Discussion

In this monocentric cohort of 76 consecutive patients with COVID 19 severe ARDS requiring ECMO, the 90-day survival was 51%. The independent predictors of 90-day survival were younger age and higher BMI.

Despite the preoccupying initial survival results displayed in the literature [9], most other important cohorts published to date find similar mortality rates as ours among ECMO COVID-19 patients, between 31 and 51% [13–16, 22–25]. When selection criteria are strict, survival rates of COVID 19 severe ARDS patients are closed to those reported in the early ECMO arm of the EOLIA trial [6] and very similar to severe ARDS patients supported by ECMO for influenza. In our cohort, as in another cohort published [26], survival was different over time, between the two waves, with 90-day survival being of 78% during the first wave and 39% during the second. This higher rate of mortality during the 2nd wave is unexplained, it could be related to a relative lower burden of the ICUs during the first wave in our region, or to highest severity of the patients who were unresponsive to dexamethasone.

We then analyzed the factors predicting 90-day mortality in our cohort, and evaluated the accuracy of previously validated ECMO survival prediction scores on COVID-19 patients.

The RESP and the PRESERVE scores were discriminating for 90-day survival, however in univariate analysis only. Another study by Supady et al. [27] evaluated these scores and found AUROC in COVID-19 patients of 0.604, 0.548 and 0.602 for the RESP, the PRESERVE and the SOFA score, respectively. Indeed, the relative homogeneity of COVID 19 severe ARDS patients (age, gender, comorbidities, organ failure involvement) could lead to less accuracy of survival scores. Thus, these scores cannot be recommended alone to indicate ECMO, but they provide helps for clinician decision. The decision to initiate ECMO should be taken on a bundle of arguments of which the scores are part, by a trained multidisciplinary team [28].

Several determinants of the prognosis of ECMO patients have been described [29]: first demographic characteristics with in the front-line, age, which has been found as a factor influencing mortality in our cohort, and in other cohorts of COVID-19 patients under ECMO [13, 14, 23, 25, 30, 31]. Second, the number of organ dysfunctions before ECMO impacts prognosis in particular acute renal failure [14] and hyperlactatemia [15]*.* Third, respiratory mechanics and management before ECMO are also classical prognostic factors. We did not find differences in respiratory mechanics between 90-day survivors and non-survivors probably due to the selection of the patients and the same phenotype with low respiratory system compliance. Of note, an earlier ECMO implantation could be associated with a better prognosis [25, 36].

Interestingly, we found that obesity was associated with 90-day survival. This result might seem surprising as obesity is a well-recognized serious risk factor for ICU admission, mechanical ventilation and mortality in COVID-19 [32–36]. Some authors hypothesized that this increased severity is may be due to adipose tissue being a reservoir for COVID-19, thus slowing down viral clearance [37, 38] and to chronic inflammatory state displayed in obese patients.

However, in non-COVID-19 patients, an “obesity paradox” has been described with an increased risk for developing pneumonia and ARDS in obese patients but no increase in mortality, or even better ICU survival rates than underweight patients in some series [39–42]. Kon et al. [41] hypothesized that this better survival in obese patients could be the consequence of the relative early lung failure due to altered respiratory mechanics (altered chest wall compliance, increase in intra-abdominal pressure, lung-volume reduction [43]), leading at the time of ECMO initiation to less lung parenchymal lesions and to faster recovery. Our results are consistent with this hypothesis with shorter time from ICU admission to intubation and ECMO in obese patients, and with a trend to better compliance with higher PEEP levels.

This study has several limitations. First it is a monocentric and partly retrospective study, making our results difficult to extrapolate to other ICUs. Second, our cohort display a relatively small number of patients, leading to difficulties of interpretation for univariate and multivariate analysis. Indeed, small samples can lead to lack of power and some risk factors could have been missed or under-estimated. Third, we did not test biological data associated with COVID-19 prognosis (CRP, d-dimers, ferritin, interleukin-6, lymphocyte ratio).

## Conclusion

In this French monocentric cohort of COVID-19 severe ARDS patients requiring ECMO, obesity was an independent factor associated with improved 90-day survival. Further studies are warranted to confirm these results.

## Supplementary Information

**Additional file 1: Figure S1.** Kaplan–Meier cumulated survival curves at day 90 since ICU admission according to obesity grades: non-obese, obesity grade 1, obesity grade 3.

## Data Availability

The datasets generated or analyzed during this study are available from the corresponding author on reasonable request.

## Abbreviations

SARS-CoV-2: Severe acute respiratory syndrome coronavirus 2
COVID-19: Coronavirus-19
ICU: Intensive care unit
MV: Mechanical ventilation
ARDS: Acute respiratory distress syndrome
ECMO: Extracorporeal membrane oxygenation
vvECMO: Veno-venous ECMO
BMI: Body mass index
RESP score: Respiratory Extracorporeal Membrane Oxygenation Survival Prediction Score [17]
PRESERVE score: PRedictiong dEath for SEvere ARDS on Veino-veinous ECMO score
SOFA score: Sequential Organ Function score
HSV: Herpes simplex virus
CMV: Cytomegalovirus
PEEP: Positive end-expiratory pressure
Pplat: Plateau pressure
AUROC: Area under the receiver operating characteristic
NO: Nitric oxide
