# Supplementary material for: Impact of obesity on survival in COVID-19 ARDS patients receiving ECMO: results from an ambispective observational cohort
Source: Ann Intensive Care. 2021 Nov 15;11:157. doi: 10.1186/s13613-021-00943-0 (PMC8591429; doi:10.1186/s13613-021-00943-0)
Supplement: Supplementary file 1 — Additional file 1: Figure S1. Kaplan–Meier cumulated survival curves at day 90 since ICU admission according to obesity grades: non-obese, obesity grade 1, obesity grade 3. [file 13613_2021_943_MOESM1_ESM.docx]

**Figure S1. Kaplan-Meier cumulated survival curves at day 90 since ICU admission according to obesity grades: non obese (blue curve), obesity grade 1 (green curve), obesity grade 3 (red curve).**

Non obese patients were defined by BMI < 30 kg/m2, obesity grade 1 by BMI ≥ 30 kg/m2 and < 35 kg/m2, obesity grade ≥ 2 by BMI ≥ 35 kg/m2.

N at Risk

Non obese

Obesity grade 1

Obesity grade ≥2

47

19

10

43

19

10

33

19

10

22

14

10

19

11

10
